# 

**Published:** 1905-04

**Authors:** 


### BOOKS RECEIVED.

The Vermiform Appendix and its Diseases. By Howard A. Kelly, A.M., M.D., Professor of Gynecology in the Johns Hopkins University, Baltimore. And E. Hurdon, M.D., Assistant in Gynecology in the Johns Hopkins University. Royal octavo, pp. xx.-887. Illustrated with 399 original illustrations, some in colors, and 3 lithographic plates. Philadelphia and London: W. B. Saunders & Company. 1905. (Price: cloth, $10.00 net; sheep or half morocco, $11.00 net.)

Gynecology. Medical and Surgical Outlines for Students and Practitioners. By Henry J. Garrigues, A.M., M.D., Gynecologist to Saint Mark's Hospital, New York. Octavo, pp xxlll.-461. With 343 illustrations. Philadelphia and London: J. B. Lippincott Company. 1905. (Price, $3.00.)

The Ophthalmic Year-Book. A Digest of the Literature of Ophthalmology for the year 1903, by Edward Jackson, A.M., M.D., Emeritus Professor of Diseases of the Eye in the Philadelphia Polyclinic. Octavo, pp. 260. With 45 illustrations. Denver: The Herrick Book and Stationery Company. 1904.

Eighteenth Annual Report of the State Board of Health of the State of New Hampshire, for the two years ending November 1, 1904. Irving A. Watson, M.D., Secretary. Concord: Rumford Printing Company.

Transactions of the American Dermatological Association. Twenty-eighth annual meeting held at Niagara Falls, N. Y., June 2 and 3, 1904. Charles J. White, M.D., Secretary. New York: The Grafton Press.

The Urine and Feces. A Practical Manual on the Urine and Feces in Diagnosis. By Otto Hensel, Ph.G., M.D., Bacteriologist to the German Hospital, New York, and Richard Weil, A.M., M.D., Pathologist to the German Hospital, New York, in collaboration with Smith Ely Jelliffe, M.D., Ph.D., Instructor in Pharmacology and Therapeutics, Columbia University, New York. Octavo, 334 pages, illustrated with 116 engravings and 10 colored plates. New York and Philadelphia: Lea Brothers & Company. 1905. (Cloth, $2.75 net.)

Saunders's Question Compends. Essentials of the Practice of Medicine. Prepared especially for students of medicine. By William R. Williams, M.D., formerly Instructor in Medicine and Lecturer in Hygiene, Cornell University; Tutor in Therapeutics, Columbia University (College of Physicians and Surgeons), New York. Duodecimo of 461 pages. Philadelphia and London: W. B. Saunders & Company. 1905. (Double number. Cloth, $1.75 net.)

The Practical Medicine Series of Year Books. Ten volumes. Issued under the general editorial charge of Gustavus P. Head, M.D., Professor of Laryngology and Rhinology in the Chicago Post-Graduate Medical School. Series 1905. Volume I. General Medicine. Edited by Frank Billings and J. H. Salisbury. Volume I. General Surgery. Edited by John B. Murphy. Chicago: The Year Book Publishers. (Price, $1.00 and $1.50; entire series, $5.50, payable in advance.)

Conservative Gynecology and Electro-Therapeutics. A Practical Treatise on Diseases of Women and their Treatment by Electricity. By G. Betton Massey, M.D., Attending Surgeon to the American Oncologic Hospital, Philadelphia. Fourth edition, revised and greatly enlarged. Illustrated with 12 original full-page chromo-lithographic plates; 12 full-page half-tone plates of photographs taken from nature, and 157 half-tone and photo-engravings in the text. Pages xvi.-468. Royal octavo. Philadelphia: F. A. Davis Company. 1905. (Cloth, beveled edges, $4.00 net.)

Lea's Series of Medical Epitomes. Edited by Victor C. Pedersen, M.D., A Manual of Medical Diagnosis. By Austin W. Hollis, M.D., Attending Physician to Saint Luke's Hospital, New York. Duodecimo, 319 pages, with 13 illustrations. New York: Lea Brothers & Company. 1905. (Cloth, $1.00 net.)

Progressive Medicine, Volume I., March, 1905. A Quarterly Digest of Advances, Discoveries and Improvements in the Medical and Surgical Sciences. Edited by Hobart Amory Hare, M.D., Professor of Therapeutics and Materia Medica in the Jefferson Medical College of Philadelphia. Octavo, 298 pages, 10 engravings and a full-page plate. Philadelphia and New York: Lea Brothers & Company. (Per annum, in four cloth-bound volumes, $9.00; in paper binding, $6.00, carriage paid to any address.)

The International Medical Annual. A Yearbook of Treatment and Practitioner's Index. Thirty-five contributors, American and Foreign. Twenty-third year. Octavo, pp. 675. New York: E. B. Treat & Company. 1905. (Price, $3.00.)

A Textbook of the Practice of Medicine. For Students and Practitioners. By Hobart Amory Hare, M.D., B.Sc., Professor of Therapeutics and Materia Medica in the Jefferson Medical College of Philadelphia. Author of A Textbook of Practical Therapeutics; A Textbook of Practical Diagnosis, etc. In one very handsome octavo volume of 1120 pages, with 129 engravings and 10 full-page plates in colors and monochrome. Philadelphia and New York: Lea Brothers & Company. 1905. (Cloth, $5.00 net; leather, $6.00 net; half morocco, $6.50 net.)

